# Comprehensive analysis of m6A regulators prognostic value in prostate cancer

**DOI:** 10.18632/aging.103549

**Published:** 2020-07-25

**Authors:** Guangjie Ji, Cong Huang, Shiming He, Yanqing Gong, Gang Song, Xuesong Li, Liqun Zhou

**Affiliations:** 1 Department of Urology, Peking University First Hospital, Institute of Urology, Peking University, National Urological Cancer Center of China, Beijing, China

**Keywords:** N6-methyladenosine, copy number variants, prostate cancer, RNA modification

## Abstract

Background: N6-methyladenosine (m6A) is the most prevalent RNA modification. While the role of m6A in prostate cancer remains unknown. We aim to measure the effects of m6A methylation regulatory genes during the development and progression of prostate cancer.

Methods: We collected transcriptome information and gene-level alteration data from The Cancer Genome Atlas datasets. The log-rank test and Cox regression model were used to examine the prognosis value of m6A methylation regulatory genes of prostate cancer.

Results: We discovered that most of m6A methylation regulators were highly expressed in aggressive prostate cancer. Univariable and multivariable Cox regression results showed that the expression of Insulin-like growth factor 2 mRNA-binding protein 3 (IGF2BP3), Heterogeneous nuclear ribonucleoproteins A2/B1 (HNRNPA2B1) and N6-adenosine-methyltransferase non-catalytic subunit (METTL14) and copy number variant of AlkB Homolog 5 (ALKBH5) were considerably associated with a recurrence-free survival of prostate cancer. Furthermore, a high level of m6A methylation in mRNA promotes the progression of prostate cancer via regulating subcellular protein localization.

Conclusion: Patients with a high level of mRNA methylation resulted from overexpression of reader proteins and methyltransferase complexes had poor survival benefits through influencing protein subcellular location in prostate cancer.

## INTRODUCTION

Prostate cancer is the leading malignant tumor in the male population worldwide, and there are no absolute effective treatments for advanced prostate cancer, especially metastatic prostate cancer and castration-resistant prostate cancer (CRPC) [1]. Although in recent years, multi-model therapy including hormone therapy, surgical prostatectomy, radiation therapy, chemical therapy, and immunotherapy have been proved to improve the prognosis of prostate cancer, biochemical recurrence, and multidrug-resistant frequently occurred in advance refractory prostate cancer and CRPC [2–4]. Thus, burrowing the cavernous molecular mechanisms should be contributed to identifying more valuable therapeutic targets.

Heretofore, there are about 172 different kinds of RNA modifications commensurate with the latest version of MODOMICS, a database of RNA modifications [5]. In particular, N^6^-methyladenosine (m6A) is one of the most extensive and exuberant internal posttranscriptional modifications in all kinds of RNA, especially in messenger RNA (mRNA) [6–8]. The formation and regulation of m6A are manipulated by a methyltransferase complex comprising three categories proteins including “readers”, “writers” and “erasers”. Proteins of YT521-B homology (YTH) domain-containing families including YTHDC1/2 and YTHDF1/2/3, Insulin-like growth factor (IGF) 2 mRNA binding families inclusive of IGF2BP2/3 and heterogeneous nuclear ribonucleoprotein (HNRNP) protein families which include HNRNPC and HNRNPA2B1 are m6A reader proteins which were able to interpret m6A methylation and provoke downstream functional signal [9–11]. “Writers” are methyltransferases that are catalyzing the formation of m6A, containing methyltransferase-like 3 (METTL3), METTL14, RNA-binding protein 15 (RBM15), RBM15B, Wilms tumor 1 associating protein (WTAP), protein virilized homolog (VIRMA) and Zinc finger CCCH domain-containing protein 13 (ZC3H13). Selectively clearing methylation in target mRNA, “erasers” function as demethylases, making up of fat mass and obesity-associated protein (FTO) and AlkB Homolog 5 (ALKBH5).

Researches have revealed that m6A methylation in mRNA is significantly associated with tumor proliferation, migration, invasion and metastasis during the process of cancer development and progression [12–14]. In lung squamous cell carcinoma, FTO stimulates cancer cell growth and metastasis with impeding cell apoptosis via controlling MZF1 expression [15]. Methyltransferase METTL3 also reinforces EGFR and TAZ expression to boost cancer progression in lung cancer [12]. High expression of METTL3 and low expression of METTL14 were associated with poor prognosis of hepatocellular carcinoma by enhancing cell proliferation and invasion [13, 16]. M6A eraser ALKBH5 stabilizes and increases target NANOG mRNA level by reducing m6A level in breast cancer stem cells. Moreover, METTL3 presented the function of promoting breast cancer progression regulated by mammalian hepatitis B X-interacting protein [17, 18]. However, the role of m6A methylation in the development and progression of prostate cancer remains questionable. Here, using integrated transcriptome and genomic analysis, we evaluated the effects of m6A methylation on the progression and survival of prostate cancer.

## RESULTS

### Expression of m6A RNA methylation regulators was correlated with prostate cancer clinical features

**Figure 1:**
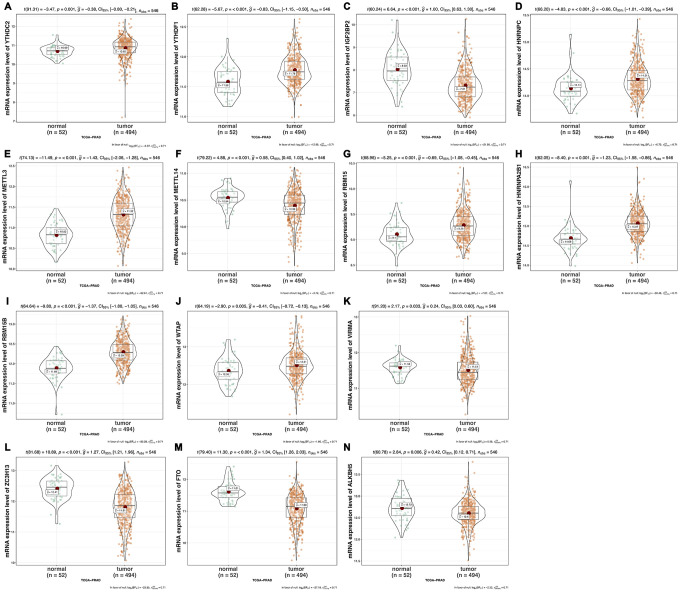
**The mRNA expression of m6A methylation regulators in normal versus tumor samples of prostate cancer respectively.** (**A**) YTHDC2; (**B**) YTHDF1; (**C**) IGF2BP2; (**D**) HNRNPC; (**E**) METTL3; (**F**) METTL14; (**G**) RBM15; (**H**) HNRNPA2B1; (**I**) RBM15B; (**J**) WTAP; (**K**) VIRMA; (**L**) ZC3H13; (**M**) FTO; (**N**) ALKBH5

**Figure 2:**
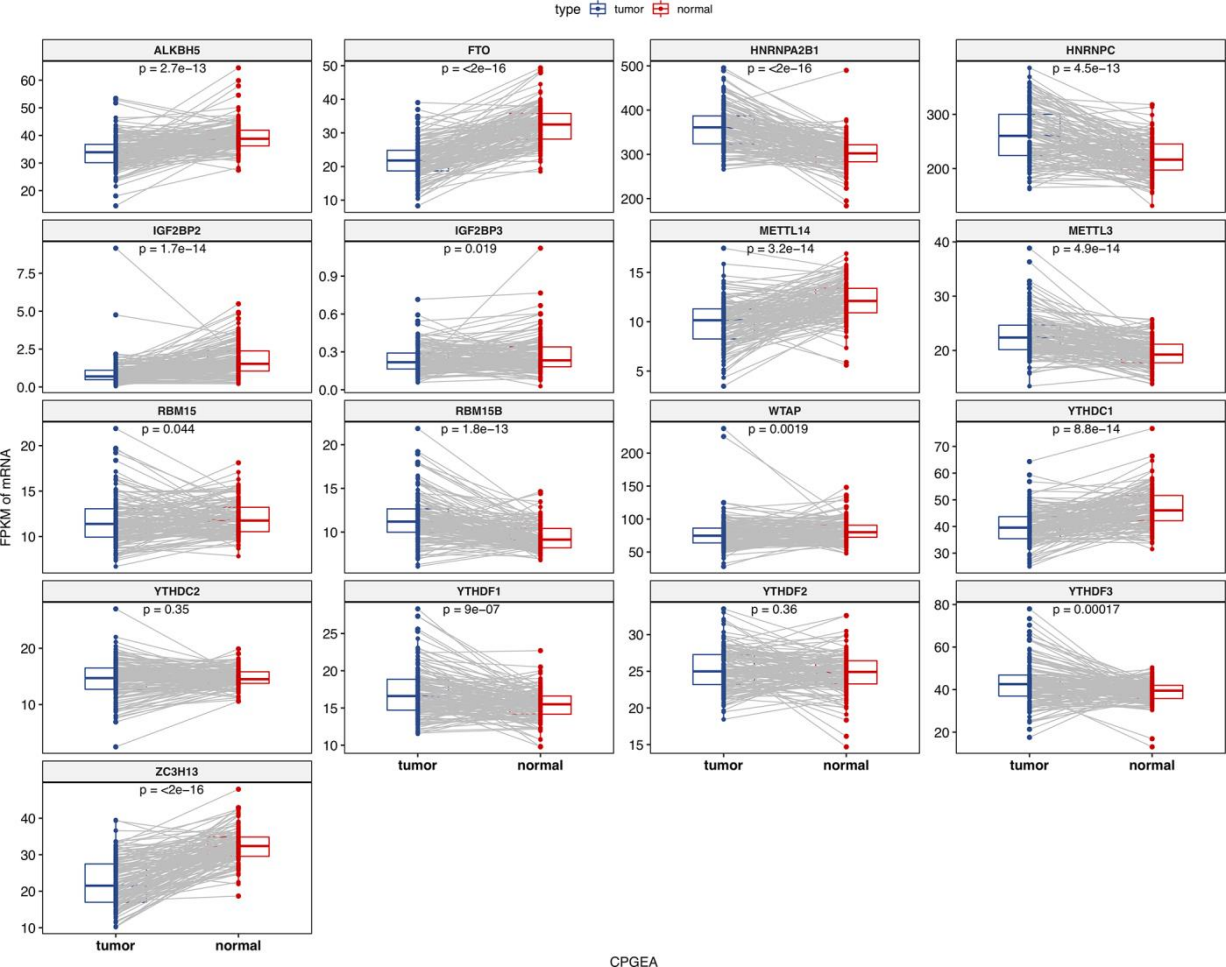
**The mRNA expression of m6A methylation regulators in paired normal and primary prostate cancer samples.**

**Figure 3:**
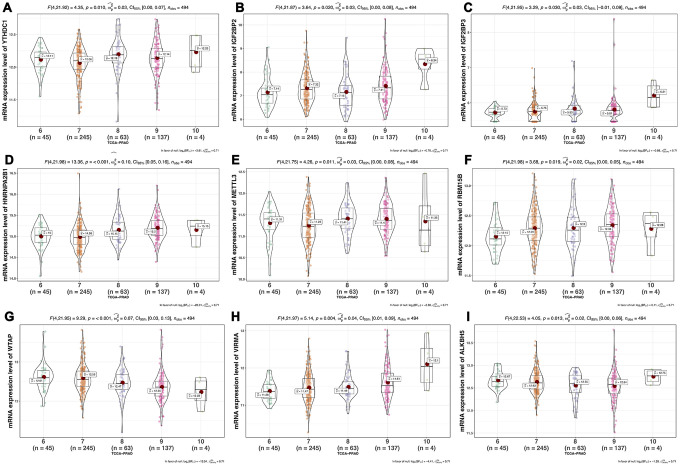
**Association between mRNA expression of m6A methylation regulators and Gleason Score of prostate cancer.**(**A**) YTHDC1; (**B**) IGF2BP2; (**C**) IGF2BP3; (**D**) HNRNPA2B1; (**E**) METTL3; (**F**) RBM15B; (**G**) WTAP; (**H**) VIRMA; (**I**) ALKBH5.

We analyzed the differences of the mRNA expression level of all the m6A RNA methylation regulators including 9 “readers”, 7 “writers” and 2 “erasers” in tumor versus normal samples of TCGA prostate cancer patients (Figure 1 and Supplementary Figure 1). Half of m6A regulators “readers” and “writers” (8/16) were highly expressed in prostate cancer than normal samples. Meanwhile, two important m6A “erasers”, FTO and ALKBH5 were significantly down-regulated in prostate cancer patients (Figure 1). Additionally, the same results were also found in 136 paired prostate cancer samples (Figure 2). Moreover, the mRNA expression of m6A regulatory genes significantly increased in patients with high Gleason Score (GS) (Figure 3).

### Survival analyses of m6A methylation regulators expression in prostate cancer patients

**Figure 4:**
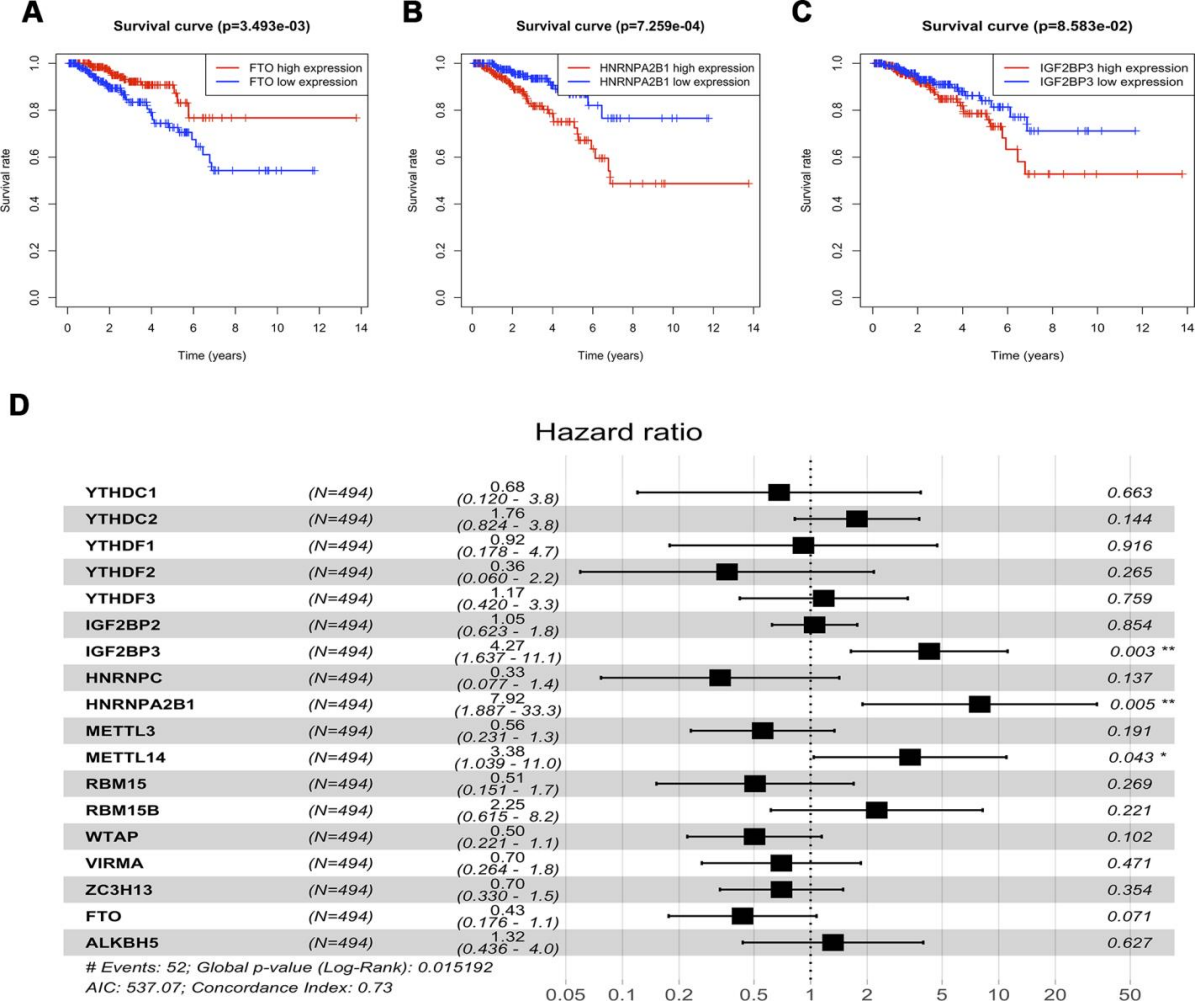
**Survival analysis of expression of m6A methylation regulators.** (**A**–**C**) Univariable cox regression analysis of there m6a methylation regulators with the significant p-value. (**D**) Multivariable cox regression analysis of all m6a methylation regulators.

**Figure 5:**
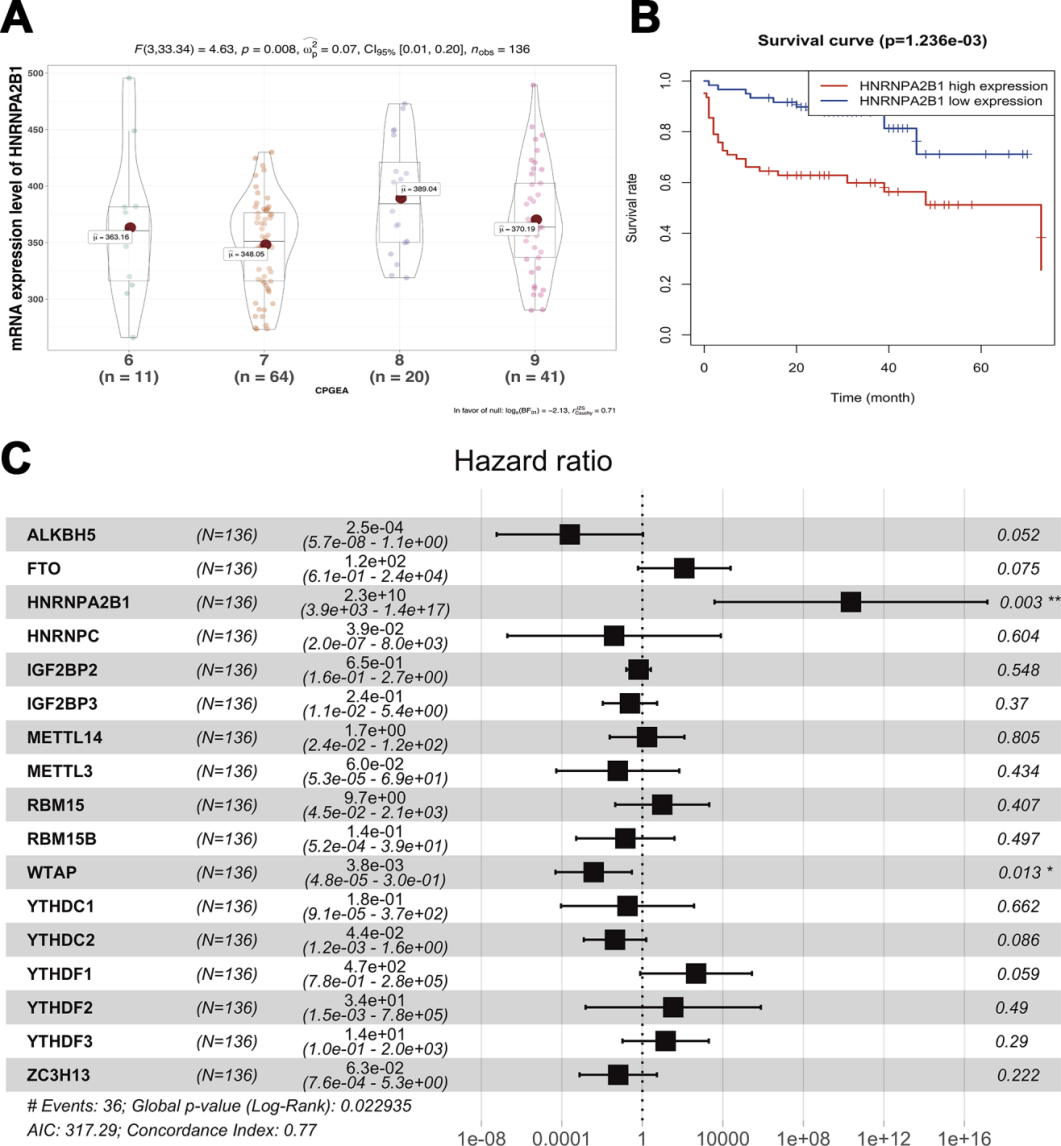
**Survival analysis of expression of m6A methylation regulators.** (**A**) correlation between expression of HNRNPA2B1 and GS; (**B**) Univariable cox regression analysis of HNRNPA2B1; (**C**) Multivariable cox regression analysis of all m6a methylation regulators.

To investigate the effects of m6A regulatory genes expression on prostate cancer recurrence-free survival (RFS), we implemented the Kaplan-Meir method and cox regression analyses. In univariate Kaplan-Meir analyses, high expression of HNRNPA2B1 and low expression level of FTO had poorer RFS of prostate cancer (Figure 4A, 4B). Similarly, IGF2BP3 (*p* < 0.05), HNRNPA2B1 (*p* < 0.05) and WTAP (*p* < 0.05) were significantly related to prostate cancer RFS (Figure 4C and Supplementary Table 1) in univariate cox regression analyses. Furthermore, multivariable Cox regression survival analysis shown that IGF2BP3 (HR = 4.27, 95%Confidence index (CI)1.637-11.1), HNRNPA2B1 (HR = 7.92, 95%CI 1.887-33.3), and METTL14 (HR = 3.38, 95%CI 1.039-11.0) were statistically meaningful risk factors of RFS in prostate cancer (Figure 4D). To validate the above results, we implement survival analyses of m6A methylation regulators expression in Chinese Prostate Cancer Genome and Epigenome Atlas (CPGEA) dataset. Consistent with results based on prostate cancer patients from TCGA, we also found that HNRNPA2B1 was not only highly expressed in patients with high GS (Figure 5A), but also significantly associated with unfavorable RFS (HR = 2.3e+10, 95%CI 3.9e+3 - 1.4e+17) (Figure 5B, 5C).

**Table 1:** Different non-silent mutations of m6A regulators in TCGA prostate cancer patients.

**PRAD sample ID**	**YTHDC1**	**YTHDC2**	**YTHDF1**	**YTHDF2**	**IGF2BP2**	**HNRNPC**	**METTL3**	**METTL14**	**RBM15**	**RBM15B**	**WTAP**	**ZC3H13**	**ALKBH5**
TCGA-CH-5791-01							*154E						
TCGA-EJ-5514-01					Q21R								
TCGA-EJ-7784-01								S399L					
TCGA-G9-6367-01									G902R				
TCGA-HC-7079-01									R59H				
TCGA-HC-7210-01						D218G							
TCGA-HC-A8CY-01												R306*	
TCGA-HC-A9TE-01												D1328H	
TCGA-HC-A9TH-01	K61fs												
TCGA-HI-7169-01									S80H				
TCGA-J4-A67T-01												K996fs	
TCGA-KK-A59V-01													R249C
TCGA-KK-A59X-01		Q670*											
TCGA-KK-A7AZ-01			G272D										
TCGA-KK-A8IA-01											R162P		
TCGA-KK-A8IB-01												D1317V	
TCGA-KK-A8IG-01								C51F					
TCGA-TP-A8TV-01									E337fs				
TCGA-VN-A88I-01												N589K	
TCGA-VP-A87D-01												R751Q	
TCGA-XK-AAIW-01		W1110L		R393Q						A792V			

### Overview of m6A methylation regulators CNVs and mutation in TCGA-PRAD dataset

**Figure 6:**
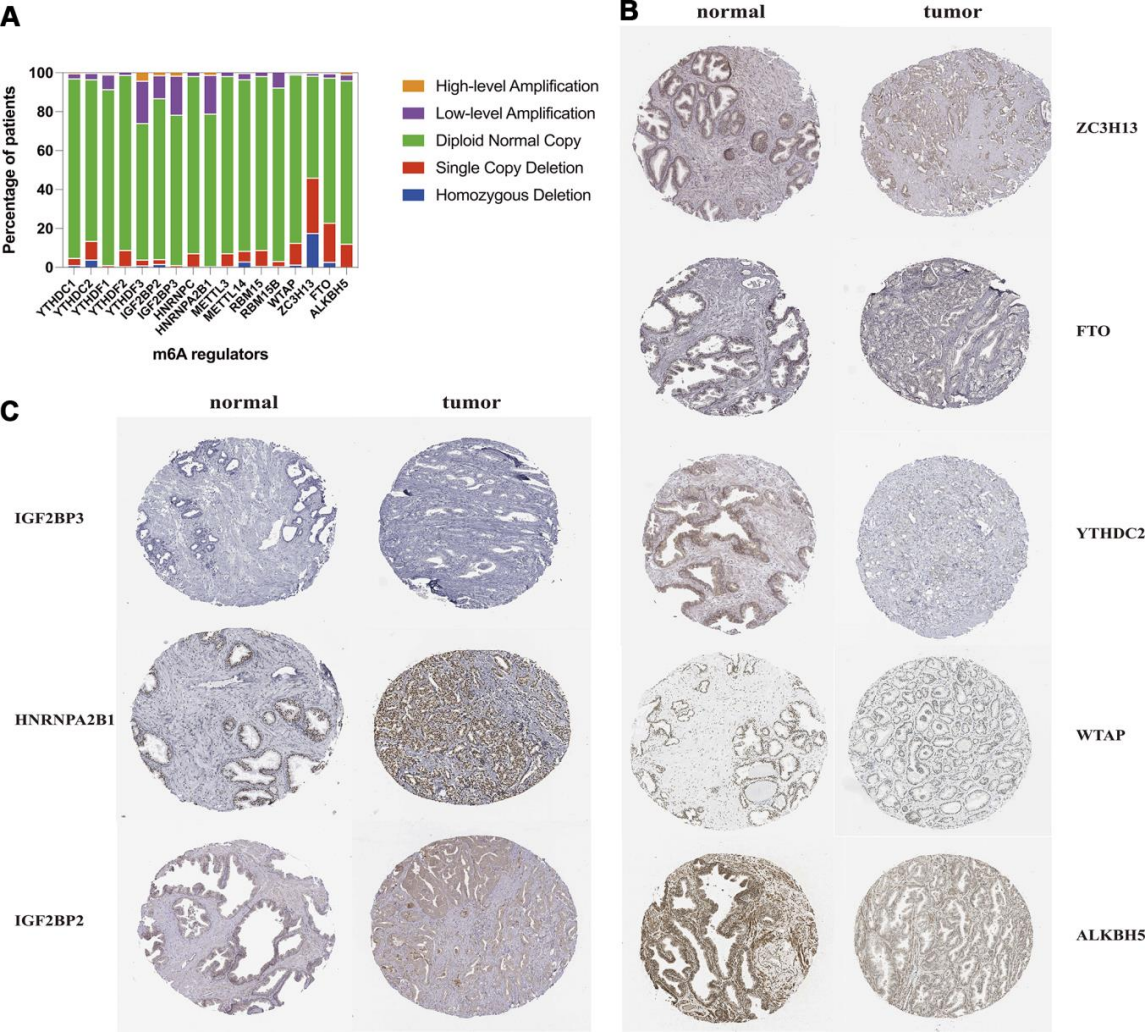
**CNV distribution and protein level of m6A methylation regulatory genes in TCGA-PRAD.** (**A**) distribution of different CNV patterns; (**B**, **C**) Immunohistochemistry images of m6A regulators from the human protein atlas database.

Among a total number of 492 cases with CNVs data in the TCGA prostate cancer dataset, about 70.2% (1213/1728) m6A methylation regulatory genes CNVs events we found were loss of DNA copy number (Supplementary Table 2 and Figure 6A). The percentage of copy number loss of ZC3H13 (46.1%) was the highest in all the m6A methylation regulators CNV loss, followed by CNV deletion percentage of FTO and YTHDC2 (Figure 6A). While the copy number gain of YTHDF3, IGF2BP3 and HNRNPA2B1 were the top 3 most frequent in all m6A regulatory genes amplification. We screened the protein level of most frequent m6A regulators in all copy number changes. We found that the copy number loss of ZC3H13, FTO, YTHDC2, WTAP and ALKBH5 decreased the protein expression in tumor samples, and meanwhile, patients with amplification of IGF2BP3, HNRNPA2B1 and IGF2BP2 presented higher expression level of these three proteins than normal tissues (Figure 6B, 6C).

Next, we screened the distribution of non-silent mutation of m6A regulators in TCGA prostate cancer patients. We observed that there were only 21 independent patients with a non-silent mutation of m6A regulatory genes among 495 cases with mutation data (Table 1).

Furthermore, the relationship between gene-level alterations including CNV and/or mutation of m6A methylation regulators and prostate cancer clinical characteristics. Obviously, the frequency of m6A regulators alterations was considerably related to higher GS (*p*=0.0001), higher tumor pathological T(*p*=0.0001), N (*p*=0.0001) stage and biochemical recurrence (*p*=0.003)(Table 2).

**Table 2:** Clinical characteristics of prostate cancer patients with or without mutation/CNV of m6A regulators.

		**with m6A regulators mutation and/or CNV**	**without 6A regulators mutation and CNV**	**P value**
Age	≤55	82	26	0.753
	>55	294	86	
Gleason Score	6	22	23	0.0001
	7	176	66	
	8	48	14	
	9	126	9	
	10	4	0	
Pathological T stage	T2a	6	7	0.0001
	T2b	7	3	
	T2c	106	57	
	T3a	130	24	
	T3b	114	17	
	T4	10	0	
	NA	3	4	
Pathological N stage	N0	265	75	0.0001
	N1	69	7	
	NA	42	30	
Biochemical recurrence	Yes	48	4	0.003
	No	278	100	
	NA	50	8	

Notes: with m6A regulators mutation and/or CNV determined as patients have mutation or CNV or mutation + CNV. Without m6A regulators mutation and CNV were patients with neither mutant nor CNV. Ambiguous variables (Nx, Mx, N/A, discrepancy and Gx) were excluded from chi-square test or non-parametric test.

### Relations between mRNA expression level and CNV of m6A methylation regulators

**Figure 7:**
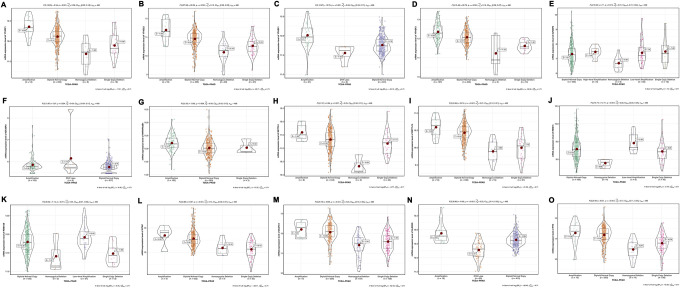
**Association between mRNA expression and different CNV patterns of m6A methylation regulators.** (**A**) YTHDC1; (**B**) YTHDC2; (**C**) YTHDF1; (**D**) YTHDF3; (**E**) IGF2BP2; (**F**) IGF2BP3; (**G**) HNRNPA2B1; (**H**) METTL3; (**I**) METTL14; (**J**) RBM15; (**K**) RBM15B; (**L**) WTAP; (**M**) ZC3H13; (**N**) ALKBH5; (**O**) FTO. Amplification: low-level amplification and high-level amplification. CNV loss: homozygous deletion and single-copy deletion.

To investigate the effects of CNV of 6A methylation regulatory genes on mRNA expression, we evaluated the mRNA expression in different CNV status. As suspected, the results have shown that gene-level amplification despite low-level or high-level was significantly associated with higher mRNA expression, while lower expression resulted from CNV loss including homozygous deletion and single-copy deletion (Figure 7). There were only YTHDF3 and HNRNPC expression did not relate to its copy number changes (Supplementary Figure 4).

### Survival analysis of m6A methylation regulators CNVs in prostate cancer patients

**Figure 8:**
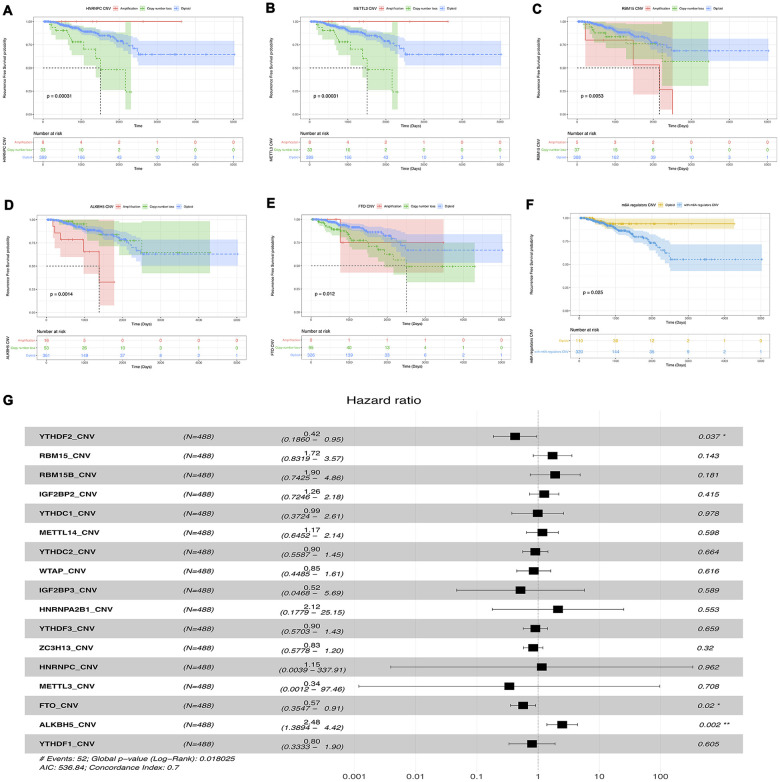
**Survival analysis of CNVs of m6a methylation regulators.** (**A**–**F**) Univariable cox regression analysis of there m6a methylation regulators with the significant p-value. (**G**) Multivariable cox regression analysis of all m6a methylation regulators.

First, univariable survival analyses were performed to examining the prognostic value of CNVs of m6A methylation regulatory genes. We found that the CNVs patterns of HNRNPC, METTL3, RBM15, ALKBH5 and FTO (one reader, two writers, and two erasers) were notably associated with RFS of prostate cancer. Specifically, copy number loss of HNRNPC, METTL3, and FTO were significantly correlated with poor survival of prostate cancer, while patients with copy number gain of RBM15 and ALKBH5 had worse RFS (Figure 8A–8F). Then, multivariable cox survival analyses were implemented to acquire more accurate information about the effects of CNVs on the prognosis of prostate cancer,. The result has shown that gene-level deletion of YTHDF3 (HR 0.42, 95% CI 0.19-0.95), FTO (HR 0.57 95% CI 0.35-0.9) and amplification of ALKBH5 (HR 2.48 95% CI 1.39-2.42) were risk factors of prostate cancer biochemical recurrence (Figure 8G).

### Bioinformatic analysis of m6A methylation risk

**Figure 9:**
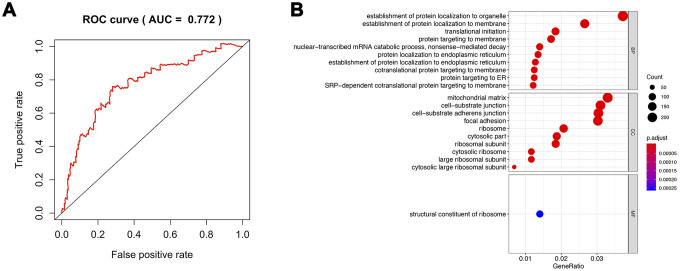
**Analysis of the pathway regulated by m6A methylation.** (**A**) ROC of all methylation regulators model. (**B**) GO analysis of differentiated genes between high risk of m6A methylation group and its low-risk group. ROC: Receiver Operating Characteristic; GO: gene ontology.

We compared the effects of mRNA expression and CNVs or mutation of m6A methylation regulators on tumor stage and prognosis of prostate cancer. We found that mRNA expression could be more valuable than gene-level alteration (Supplementary Figure 6). Furthermore, the area under receivers (AUC) of the multivariable Cox survival regression based on m6A regulators expression reached 0.772 (Figure 9A). Therefore, we classified the prostate cancer patients into high m6A risk groups and low m6A risk groups according to multivariable cox regression. Then, gene ontology analysis was performed between two groups. The results presented that protein location was significantly associated with m6A methylation regulation (Figure 9B).

## DISCUSSION

Considering the regulation of m6A methylation was complicated and multidimensional, it is impossible that all m6A methylation regulatory genes presented the same trend in prostate cancer. We discovered that apart from 4 “readers” (YTHDC1, YTHDF2, YTHDF3 and IGF2BP3) whose mRNA expression level were insignificant differences in tumor and normal samples, mRNA expression of two “readers” (IGF2BP2 and METTL14) and two “writers” (VIRMA and ZC3H13) were lower in prostate cancer sites than in normal samples. Moreover, high mRNA expression of WTAP as a “writer” was associated with low GS. This discordance or contradiction illustrated that the biological effects of different m6A methylation regulators on the development and progression of prostate cancer could be different from each other. In this study, we found HNRNPA2B1, METTL3, and RBM15B were overexpressed not only in tumor samples but also in high GS prostate cancer patients. The role of HNRNPA2B1, METTL3, and RBM15B may play an oncogene in prostate cancer. Currently, there were few pieces of research related to HNRNPA2B1 an RBM15B in prostate cancer. Similar to our research, Cai et al. found that METTL3 was overexpressed and promotes tumor proliferation and invasion in prostate cancer [19].

Our study combined mRNA expression level and gene-level CNVs and mutation alteration analysis of m6A methylation regulators in prostate cancer. Although the percentage of m6A regulators mutations in TCGA prostate cancer cohort is only 4.1%, the biological effects of these mutations have been proofed to be important during tumor progression. A mutation in METTL14 could promote cancer cell proliferation through decreasing m6A level and activating AKT signaling pathway [14]. There are few pieces of researches focused on mutations in m6A-related genes in prostate cancer, but in AML, mutations in m6A writers such as METTL3, METTL14 and WTAP can enhance progression of leukemia, conferring unfavorable survival outcomes [20–23]. As is well-known, CNVs were significantly associated with mRNA expression. Specifically, copy number gain could lead to overexpression of genes, and copy number loss decreases the transcription of genes. Beside of YTHDF2 and HNRNPC among all m6A methylation regulators, CNV of most of them were highly related to their mRNA expression level. Integrated the survival analysis results of mRNA expression and CNV, high expression and gene-level amplification of FTO were importantly associated with a favorable survival benefit. However, the results of multivariable cox survival regression analyses according to mRNA expression and CNV of m6A methylation regulators were totally different. Furthermore, combined with clinical features of prostate cancer, the differences between the final results of these two models still existed. We presented IGF2BP3, HNRNPA2B1 and METTL14 were significantly related to RFS of prostate cancer based on mRNA expression information, while in the CNV-based regression model, YTHDF2, FTO, and ALKBH5 were notably associated with prognosis of prostate cancer. Interestingly, there were no apparent differences in the mRNA expression of YTHDF2 in different types of YTHDF2 copy number alterations. It implied that the relationships of transcriptional activity and gene-level CNV is indirect and complicated.

Importantly, IGF2BP3, HNRNPA2B1, and METTL14 were significantly associated with RFS of prostate cancer in multivariable cox regression established on mRNA expression of m6A methylation regulators. To confirm our funding, we added critical clinical characteristics such as diagnosed age, GS, tumor T stage and N stage, the above three m6A methylation regulators (IGF2BP3, HNRNPA2B1, and METTL14) were still remarkably related with prognosis of prostate cancer. Similarly, the previous study has reported that IGF2BP3 was highly expressed in metastatic prostate cancer patients and independently correlated to worse cancer-specific survival [24–26]. However, the role of mRNA methylation in prostate cancer patients with IGF2BP3 overexpression had not been mentioned in the above studies. In other types of human carcinoma, IGF2BP3 was also a valuable prognosis marker. Patients with higher expression of IGF2BP3 showed longer metastasis-free survival and overall survival in renal-cell carcinoma [27]. The overexpression of IGF2BP3 was related to poor disease-specific survival of gastric cancer, regulated by miRNA-34a [28]. In upper tract urothelial carcinoma, patients with up-regulated IGF2BP3 presented significantly poorer RFS, cancer-specific survival and overall survival [29]. As a member of the critical family of m6A readers that identify the consensus GG(m6A)C sequence, IGF2BP3 participates in thousands of mRNA posttranscriptional modification through enhancing the stability and accommodation of its target mRNA (like MYC) in cancer biology [30].

Acting as a nuclear m6A reader, HNRNPA2B1 regulates alternative splicing of exons importantly related to METTL3, through targeting to nuclear RNAs containing RGm6AC sites *in vivo* and *vitro* [31]. Furthermore, HNRNPA2B1 promotes the processing of pri-miRNAs by interacting with DGCR8, a critical component of pri-miRNA microprocessor complex. And HNRNPA2B1 was identified as a key regulator that stabilizes a high number of its target mRNAs through mass spectrometry and binding sites studies [32]. Knockdown of HNRNPA2B1 using small hairpin RNAs impaired tumor viability and proliferation via inactivating c-Akt signaling pathway in KRAS phosphorylation-dependent pancreatic ductal adenocarcinoma cells [33]. In ovarian cancer, HNRNPA2B1 ameliorated tumor growth through binding and stabilizing Lin28B mRNA resulting in poor survival [34]. High expression of HNRNPA2B1 progressed the proliferation of prostate cancer in elevating endogenous beta-catenin mRNA translation and nuclear localization [35].

METTL14 plays a crucial role in m6A methylation modification of RNA [36]. It is reported that METTL14 recognized and bound directly H3 trimethylation at Lys36 to increase m6A abundance of the transcriptome [37]. Highly expressed in normal hematopoietic stem cells, METTL14 impeded myeloid differentiation and showed oncogenic function via m6A modification of its target mRNA in acute myeloid leukemia [23]. In the METTL14 knock out mouse model, embryonic neural stem cells presented sharply depressed growth and differentiation by regulating histone modifications [38]. While in some other types of carcinoma, METTL14 exhibited the role of tumor suppressor by regulating m6A modification [13, 39, 40]. In this study, we first found METTL14 was highly expressed in the normal samples in prostate cancer patients, and insignificantly associated with GS and RFS of prostate cancer. However, the multivariable cox regression model with or without clinical features demonstrated METTL14 considerably related to the survival benefit of prostate cancer, which suggested the m6A modification controlled by METT14 was predisposed to various factors and complicated.

This study was the first time described in detail the whole landscape gene-level and mRNA expression level of m6A methylation regulators in prostate cancer, and their effects on the prognosis of prostate cancer. Patients with a high level of mRNA methylation resulted from overexpression of m6A methylation regulators of “readers” and “writers” had more inferior survival benefits through influencing protein subcellular location in prostate cancer. These findings provide sustenance for exploring further epigenetic regulation mechanisms in prostate cancer.

## MATERIALS AND METHODS

### Data collection and process

The Cancer Genome Atlas (TCGA) prostate adenocarcinoma database was downloaded from the University of California Santa Cruz Xena (https://xena.ucsc.edu/), including gene expression RNAseq, copy number (gene-level), non-silent somatic mutation data and phenotype information. Prostate adenocarcinoma thresholded gene-level copy number variation (CNV) was estimated using the GISTIC2 method. Finally, we identified 494 prostate adenocarcinoma patients with complete mRNA expression and clinical information, and 488 prostate patients with CNV and pathological data. Another dataset used in this study was the Chinese Postate Cancer Genome and Epigenome Atlas (CPGEA) downloaded from http://www.cpgea.com/ [41].

### Statistical analysis

Standard student t-test and one-way analysis of variance (ANOVA) were used to compare the mRNA expression level of m6A methylation regulators in different clinical subgroups and CNV phenotypes in prostate adenocarcinoma. Chi-square tests were carried out to compare the distribution of age, Gleason Score, T and N stage in “with m6A regulators mutation and/or CNV” and “without m6A regulators mutation and CNV” group.

The Kaplan-Meier method with a two-sided log-rank test and univariate and multivariate Cox regression analyses were performed to find the prognostic value of m6A regulators in expression and gene-level alteration. All statistical analyses and data visualizations were based on R v3.6 (https://www.r-project.org).

## Supplementary Material

Supplementary Figures

Supplementary Tables
