# Supplementary material for: Comprehensive analysis of m6A regulators prognostic value in prostate cancer
Source: Aging (Albany NY). 2020 Jul 25;12(14):14863–84. doi: 10.18632/aging.103549 (PMC7425456; doi:10.18632/aging.103549)
Supplement: Supplementary Figures [file aging-12-103549-s002..pdf]

SUPPLEMENTARY FIGURES

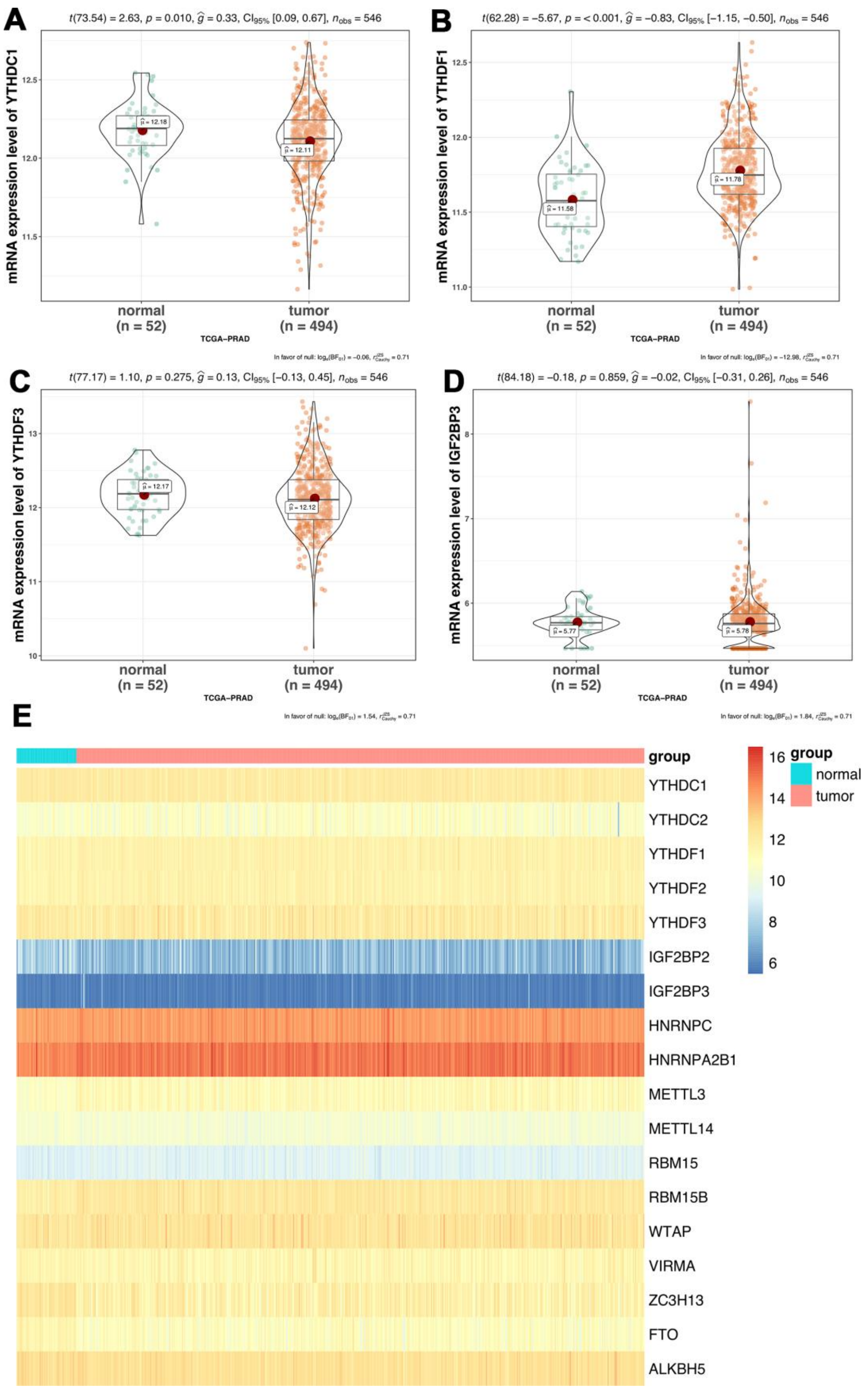

Supplementary Figure 1. The mRNA expression of m6A methylation regulators in normal versus tumor samples of prostate cancer respectively. (**A**–**D**) YTHDC1, YTHDF2, YTHDF3, IGF2BP3. (**E**) Heatmap of m6A methylation regulatory genes expression in normal and tumor samples.

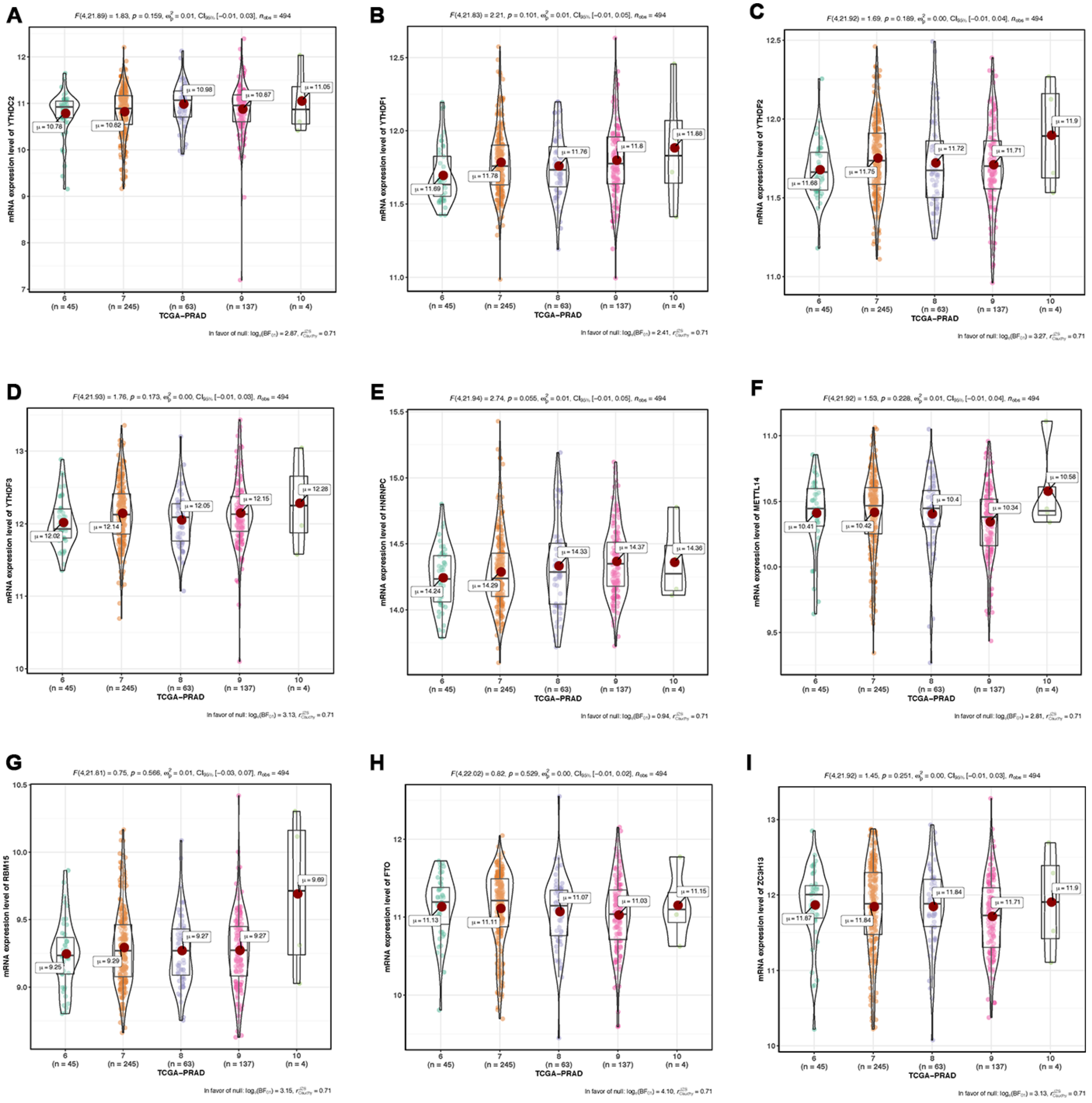

Supplementary Figure 2. (**A**–**I**) YTHDC2, YTHDF1, YTHDF2, YTHDF3, HNRNPC, METTL14, RBM15, FTO, ZC3H13.

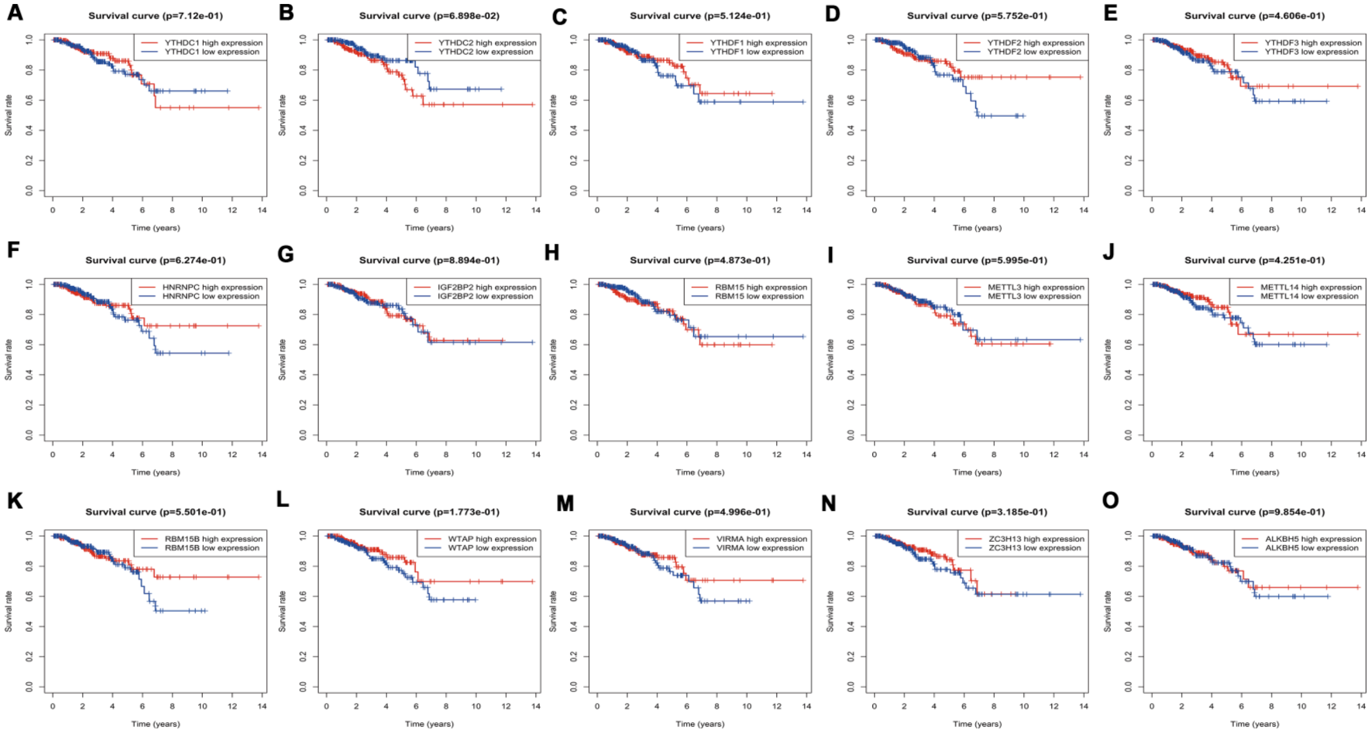

Supplementary Figure 3. (**A**–**O**) YTHDC1, YTHDC2, YTHDF1, YTHDF2, YTHDF3, HNRNPC, IGF2BP2, RBM15, METTL3, METTL14, RBM15B, WTAP, VIRMA, ZC3H13, ALKBH5.

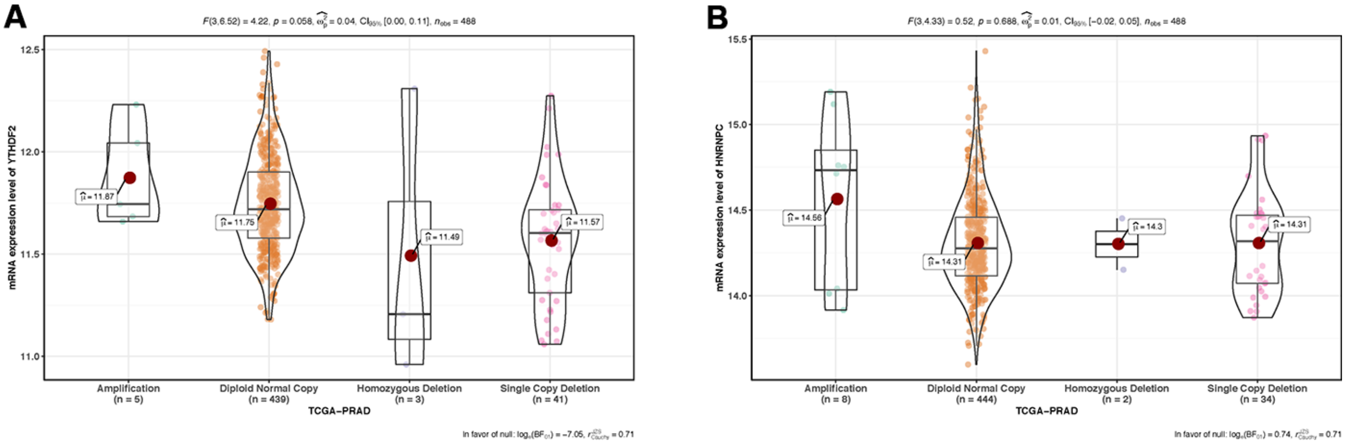

Supplementary Figure 4. (**A**) YTHDF2; (**B**) HNRNPC. Amplification: low-level amplification and high-level amplification. CNV loss: homozygous deletion and single-copy deletion.

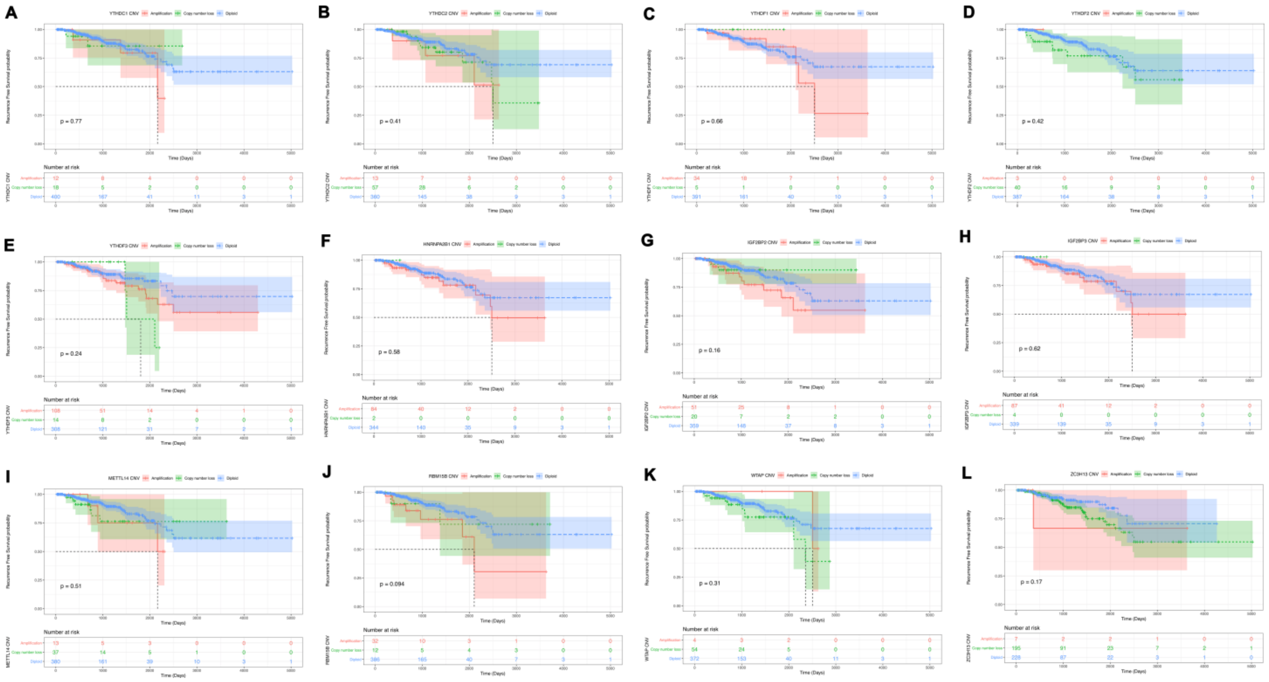

Supplementary Figure 5. (**A**–**L**) YTHDC1, YTHDC2, YTHDF1, YTHDF2, YTHDF3, HNRPA2B1, IGF2BP2, IGF2BP3, METTL14, RBM15B, WTAP, ZC3H13.

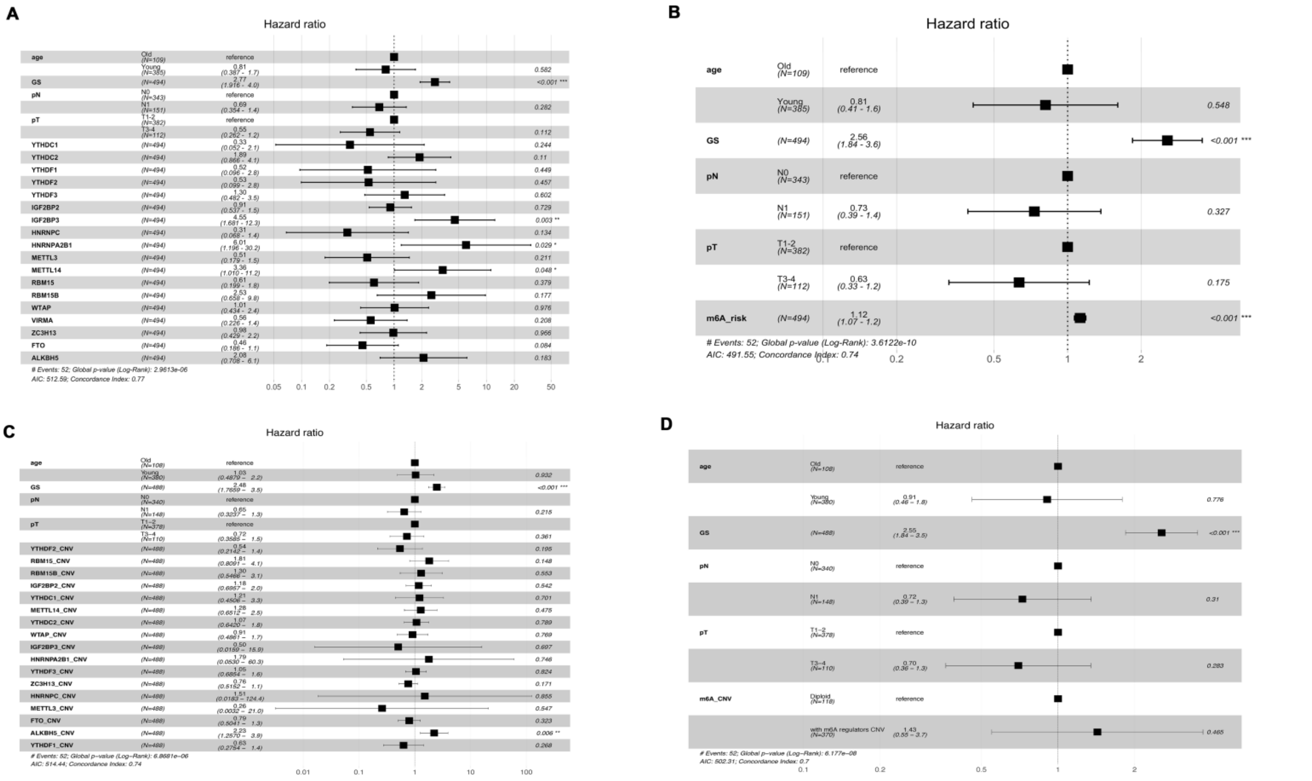

Supplementary Figure 6. Multivariable cox survival regression analysis of m6A methylation regulators. (**A**) Forest plot of Cox regression based on the mRNA expression of every m6A regulators and clinical characteristics. (**B**) Forest plot of Cox regression based on combined m6A risk and clinical information. (**C**) Forest plot of Cox regression based on the CNV pattern of every m6A regulators and clinical characteristics. (**D**) Forest plot of Cox regression based on combined m6A CNV and clinical information.

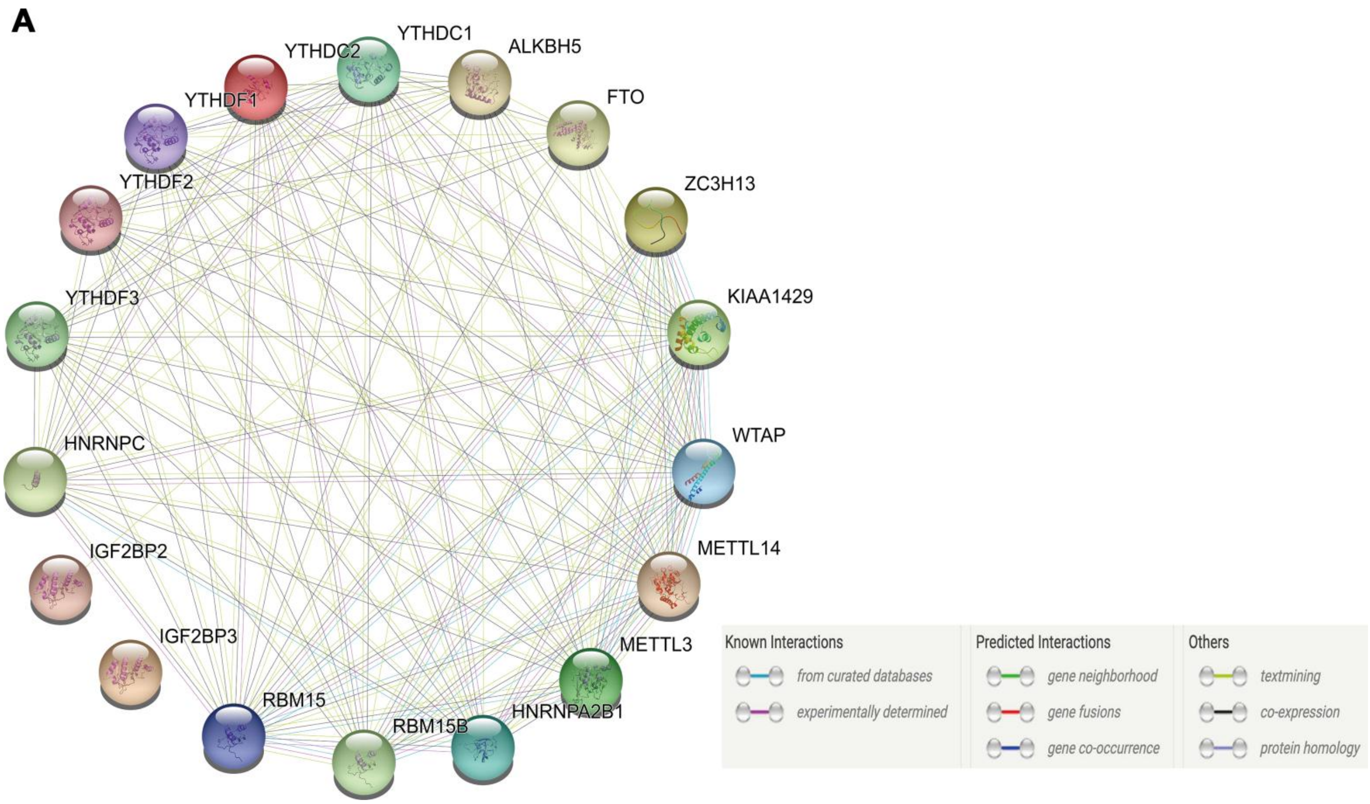

Supplementary Figure 7. Protein-protein interaction network of all m6A regulators from the STRING database.
