# Supplementary material for: Comprehensive analysis of m6A regulators prognostic value in prostate cancer
Source: Aging (Albany NY). 2020 Jul 25;12(14):14863–84. doi: 10.18632/aging.103549 (PMC7425456; doi:10.18632/aging.103549)
Supplement: Supplementary Tables [file aging-12-103549-s001..pdf]

SUPPLEMENTARY TABLES

Supplementary Table 1. Univariable Cox regression analysis of the mRNA expression of every m6A regulators separately.

m6A regulators	HR	z	*p*-value
IGF2BP3	1026.46744	3.07198481	0.002126405
HNRNPA2B1	593533.11	2.79316328	0.005219534
WTAP	0.00063203	-2.1648639	0.030398107
METTL3	55.0593509	1.40830364	0.159041179
FTO	0.047653	-1.3682079	0.171247014
YTHDC2	17.0374393	1.26930186	0.204333423
ALKBH5	0.02075067	-0.9389564	0.34775315
ZC3H13	0.15060789	-0.9236734	0.355656374
RBM15B	44.4700655	0.90494594	0.365494028
YTHDF2	0.01190774	-0.8552777	0.39239742
VIRMA	8.79632016	0.72681565	0.467338895
RBM15	0.20936779	-0.4446332	0.656584833
HNRNPC	0.09684105	-0.4369219	0.662167973
YTHDF1	0.17517754	-0.3242348	0.745760274
YTHDC1	4.40345227	0.28655265	0.774454875
YTHDF3	1.80704077	0.20507624	0.837512558
IGF2BP2	0.9019237	-0.0806378	0.935729981
METTL14	0.94128837	-0.0174269	0.986096066

Supplementary Table 2. Different CNV patterns presented in prostate cancer patients from TCGA.

		Homozygous deletion	Single copy deletion	Diploid normal copy	Low-level amplification	High-level amplification	Percentage
Reader	YTHDC1	6	18	454	13	2	0.07910751
	YTHDC2	20	47	409	16	0	0.16869919
	YTHDF1	1	5	445	37	4	0.09552846
	YTHDF2	3	41	443	5	0	0.0995935
	YTHDF3	5	15	345	107	20	0.29878049
	IGF2BP2	9	12	407	58	6	0.17276423
	IGF2BP3	1	5	380	99	7	0.22764228
	HNRNPC	2	34	448	8	0	0.08943089
	HNRNPA2B1	0	3	386	98	5	0.21544715
Writer	METTL3	3	33	448	8	0	0.08943089
	METTL14	15	27	434	16	0	0.11788618
	RBM15	4	40	440	8	0	0.10569106
	RBM15B	3	13	439	37	0	0.10772358
	WTAP	7	55	426	4	0	0.13414634
	ZC3H13	87	140	258	7	0	0.47560976
Eraser	FTO	14	99	367	11	1	0.25406504
	ALKBH5	1	59	413	15	4	0.16056911
